# Adherence to a Lifestyle Exercise and Nutrition Intervention in University Employees during the COVID-19 Pandemic: A Randomized Controlled Trial

**DOI:** 10.3390/ijerph18147510

**Published:** 2021-07-14

**Authors:** Guillermo García Pérez de Sevilla, Olga Barceló Guido, María de la Paz De la Cruz, Ascensión Blanco Fernández, Lidia B. Alejo, María Montero Martínez, Margarita Pérez-Ruiz

**Affiliations:** 1 Department of Physiotherapy, Faculty of Sport Sciences, Universidad Europea de Madrid, 28670 Madrid, Spain; 2 Faculty of Sport Sciences, Universidad Europea de Madrid, 28670 Madrid, Spain; olga.barcelo@universidadeuropea.es (O.B.G.); lidia.brea@universidadeuropea.es (L.B.A.); margarita.perez@universidadeuropea.es (M.P.-R.); 3 Medical Service, Universidad Europea de Madrid, 28670 Madrid, Spain; mariadelapaz.delacruz@universidadeuropea.es; 4 Faculty of Biomedical and Health Sciences, Universidad Europea de Madrid, 28670 Madrid, Spain; ascension.blanco@universidadeuropea.es (A.B.F.); mariamonteromartinez11@gmail.com (M.M.M.)

**Keywords:** lifestyle, intervention, physical exercise, Mediterranean diet, COVID-19 pandemic, telematic, adherence, workplace

## Abstract

Healthy lifestyles should be encouraged in the workplace through the occupational health teams of the companies. The objective of the present study was to evaluate the adherence to a lifestyle intervention carried out in university employees during the COVID-19 pandemic and its impact on health-related quality of life (HrQoL). A randomized controlled trial following the CONSORT guidelines was performed, consisting of three supervised interventions lasting for 18 weeks: an educational intervention on healthy habits, a nutritional intervention, and a telematic aerobic and strength exercise intervention. Lifestyle and HrQoL were analyzed six months post-intervention to assess adherence. Twenty-three middle-aged participants completed the study. The intervention group significantly improved their lifestyle according to the Health Promoting Lifestyle Profile II questionnaire, especially in the categories of Health Responsibility, Physical Activity, and Nutrition, with a large effect size. Sitting time was reduced by 2.5 h per day, with a moderate effect size. Regarding HrQoL, the intervention group showed a clinically significant improvement in the Physical Component Summary. Despite the lockdown and the mobility restrictions caused by the COVID-19 pandemic, this intervention performed on university employees achieved adherence to a healthier lifestyle and improved their HrQoL, which is of great clinical relevance.

## 1. Introduction

The COVID-19 pandemic has affected and overwhelmed our healthcare systems and our lifestyles in an unprecedented way. Consequently and pending a massive and effective vaccination, social distance and mobility restrictions are the main preventive measures to stop the transmission of SARS-CoV-2 [1]. To comply with these measures we have reorganized our society and our lifestyle during this pandemic. As a negative impact, the number of hours per day sitting has increased between 23.8–28.6% [2,3,4,5,6], and physical activity (PA) levels have decreased by 28.8–38% [4,6,7], Dietary habits have also been affected. Even if the adherence to the Mediterranean diet has increased slightly [6,7,8], the prevalence of snacking and the consumption of alcoholic and carbonated drinks has also increased [4,7].

Increased sedentary behavior predisposes to developing metabolic, cardiovascular, pulmonary, neurological, musculoskeletal, psychological, and oncological diseases [9]. Possible mechanisms are increasing oxidative stress and inflammation caused by an increase in visceral fat and a decrease in muscle mass [10].

Every hour of sedentary behavior a day affects our health [11,12,13], establishing 6–8 h a day of sitting as a cut-off point to consider a risk factor for increased mortality [14,15,16,17]. This threshold has been exceeded by workers during the pandemic, due to the teleworking situation, according to various authors in different countries [2,3,6]. In addition, increased sedentary behavior attenuates the physiological adaptations induced by PA [13,18,19,20], so even people who maintained their exercise routine have been affected by mobility restrictions and the new lifestyle caused by the pandemic [18].

If the decrease in PA (38%) and the increase in sedentary behavior (28.6%), reported by Ammar et al. [4], were maintained over time, as estimation of the cases of type 2 diabetes and the mortality from all causes worldwide would increase between 7.2–9.6% and 9.4–12.5%, respectively [21]. Lower levels of PA have also affected mental health, increasing the prevalence of anxiety and depression, symptoms that can, in turn, affect the adoption of unhealthy lifestyles [22].

PA, through epigenetic modulation [23], influences the prevention and development of numerous non-communicable diseases (NCDs) [24]. In this sense, there is a dose-response relationship regarding the effects of physical exercise in preventing NCDs [25]. The WHO published general recommendations for promoting PA worldwide in 2020 to achieve primary prevention of NCDs [26]. Regular practice of both aerobic and strength exercise is associated with a reduction in mortality by 21–40%, depending mainly on exercise volume and exercise intensity [11,27].

Observational and epidemiological studies associate adherence to the Mediterranean diet with a protective effect against cardiometabolic diseases [28,29,30]. However, more randomized controlled trials analyzing the effectiveness of Mediterranean diet interventions are lacking [31,32,33,34].

Healthy lifestyles should be encouraged in all age groups since health risk factors develop from childhood. The work environment covers an age group with risk factors, but generally without declared disease, where it is easy for the occupational health teams of the companies to act due to the high number of hours spent in the workplace. In this respect, the WHO global action plan on workers’ health establishes that lifestyle interventions should be carried out within the workplace [35].

Exercise interventions carried out in the work environment consisting of supervised aerobic and strength exercise, lasting at least four months and at least two weekly sessions, achieve a clinically significant decrease in fat mass and an increased muscle mass [36,37,38,39]. One of these studies reported an improvement in systolic blood pressure [37]. The studies that consist of both exercise and nutrition interventions report significant reductions in body weight and waist circumference [40,41].

According to a recent meta-analysis, interventions carried out in the workplace to increase PA levels achieve increases of 210 metabolic equivalent tasks (METS)-min per week [42]. Regarding interventions to reduce sedentary behavior within the working day, studies of high methodological quality report reductions of 0.5–1.5 h per day of sitting time [43]. On the other hand, a few studies on Mediterranean diet interventions of high methodological quality have been performed at the workplace, without managing to improve the adherence to this type of diet [40,44].

Most of the studies that provide nutrition and exercise interventions in the obese population successfully achieve their objectives but fail to maintain adherence to the new habits, losing effectiveness in the long term, generally after six months [45]. The same occurs with telematic interventions that assess PA levels [46] or sedentary behavior [47] with long-term follow-up. Only one study has reported an increase in PA levels with the maintenance of this achievement six months after the intervention [48]. For this reason, we analyzed adherence to healthy habits six months after the intervention.

No randomized clinical trial has been found reporting a telematic intervention at the workplace that manages to increase PA levels [49,50] and to date, none carried out during the COVID-19 pandemic lockdown is described in the literature. Only one observational study has been published, in which they did not perform an intervention [6].

Objective: The objective of the present study was to evaluate the adherence to a lifestyle exercise and nutrition intervention carried out in university employees during the COVID-19 pandemic, and its impact on health-related quality of life (HrQoL).

## 2. Methods

### 2.1. Study Design

A randomized controlled trial with a parallel group design consisting of an intervention arm and a control arm was developed following the CONSORT guidelines.

### 2.2. Participants

Participants were recruited by nonprobabilistic convenience sampling from the occupational medical service of the Universidad Europea in December 2019. An informative meeting of the program was held, after which people interested in participating in the study filled out the questionnaires Global Physical Activity Questionnaire (GPAQ), University of Rhode Island Change Assessment Scale (URICA) and Mediterranean Diet Adherence Score (MEDAS), to see if they met the inclusion criteria.

The inclusion criteria to participate in the study were the following: (1) Working-age adults; (2) Failure to comply with 2010 WHO physical exercise recommendations [51]; (3) Score ≤ 9 in the MEDAS questionnaire, which means low or medium adherence to the Mediterranean diet [52]; (4) being in the contemplation stage according to the URICA questionnaire [53].

The exclusion criteria were: (1) Having a diagnosed chronic disease that the medical service considers as a contraindication to physical exercise; (2) Having musculoskeletal injuries that make it impossible to perform physical exercise.

### 2.3. Sample Size

To calculate the sample size, we used data from a pilot study with a mixed design of two repeated measures and two groups. The primary variable was the effect on lifestyle using the Health Promoting Lifestyle Profile II (HPLP II), with an alpha error of 0.05 and a beta error of 0.2. Using the G-Power v.3.1 software (Erdfelder et al., Kiel, Germany), the resulting *n* needed to cover our objective was 22 subjects, so we sampled 24 subjects to compensate for a probable 10% loss of sample.

### 2.4. Randomization

Before randomization, the sample was matched by BMI and age, taking the median as a reference to achieve a homogeneous distribution in both groups. Subsequently, the randomization of the participants was carried out with the random function of Microsoft Office Excel (Microsoft Corporation, Redmond, WA, USA), being assigned to the control group (CG) or the intervention group (IG). All the participants read and signed the corresponding, specific, informed consent form before being part of this investigation. Then, a code was assigned to each participant. The correspondence table between the personal identification data and the code was kept exclusively by the lead researcher and was kept separate from the rest of the study data. There was no blinding for the participants, the therapists, the evaluators, or the researcher who analyzed the statistical data.

### 2.5. Variables

#### 2.5.1. Lifestyle

The HPLP II questionnaire was filled out to analyze the lifestyle. It consists of 52 items that are answered as N (never), A (sometimes), M (frequently), and R (routinely). It consists of 6 categories: Nutrition (9 items), Physical activity (8 items), Health responsibility (9 items), Stress management (8 items), Interpersonal relationships (9 items), and Spiritual growth (9 items) [54,55]. The minimum score is 52, and the maximum is 208. A score of 52–90 is considered a low score, and therefore an inadequate lifestyle, 91–129 is considered moderate and an improvable lifestyle, 130–168 is a good lifestyle, and 169–208 is an excellent lifestyle [56].

Adherence to the Mediterranean diet was assessed through the MEDAS questionnaire to complement the analysis of the lifestyle. The MEDAS consists of 14 items, of which each adds 0 or 1 point. A score ≥ 10 is considered high adherence, a score of 8–9 is considered medium adherence, and a score ≤ 7 is considered low adherence. High adherence to the Mediterranean diet is considered a strong protector against cardiovascular diseases [52]. In addition, a 1-point improvement on this questionnaire is associated with a 6% decrease in all-cause mortality [57].

To evaluate another component of lifestyle, PA levels and sedentary behavior were analyzed using the questionnaire GPAQ [16,58]. The GPAQ consists of a question that analyzes the daily hours that the subject remains seated, and of 15 questions that assess the PA levels in the categories of work, travel, and leisure time, estimating energy expenditure in weekly METS-min and classifying subjects into three categories [26]: Category 1: Low. This is the lowest PA level, which means not meeting the criteria of categories 2 or 3. Category 2: Moderate. Individuals perform at least 20 min of vigorous activity ≥ three days per week, or at least 30 min of moderate-intensity PA ≥ five days per week, or 600 METs-min weekly of moderate-intensity PA spread over at least five days per week. Category 3: High. Individuals perform 1500 METs-min per week of vigorous-intensity PA spread over at least three days per week, or 3000 METs-min per week of moderate to vigorous-intensity PA spread over the seven days of the week.

#### 2.5.2. Health-Related Quality of Life

To assess HrQoL, the participants filled out the Short Form 36 Health Survey Questionnaire v2 (SF-36) [59]. It consists of 36 items, which gives a score from 0 (worst self-perception of HrQoL) to 100 (best self-perception of HrQoL) in 8 sections: Physical Function, Role Physical, Bodily Pain, General Health, Vitality, Social Functioning, Role Emotional, and Mental health.

The eight sections are regrouped into two main components: Physical Component Summary and Mental Component Summary. A 4-point increase in one of these two components, after an intervention, is considered clinically relevant, in a healthy adult population [60].

#### 2.5.3. Anthropometric Variables

Height (cm, Ano Sayol SL height rod, Barcelona, Spain) and weight (kg, Asimed T2 scale, Barcelona, Spain) were measured. Then, by dividing the weight in kg by the height in meters squared, the body mass index (BMI, in kg/m^2^) was calculated.

### 2.6. Lifestyle Intervention

**Figure 1:**
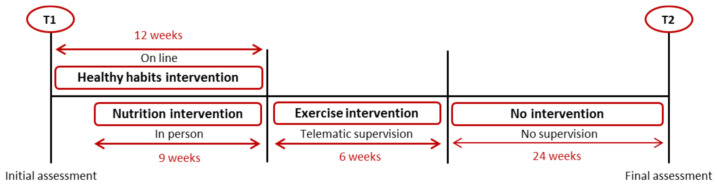
Organization of the intervention over time of a lifestyle intervention during the COVID-19 pandemic.

This program consisted of three interventions (Figure 1). First, an educational intervention on healthy habits was carried out in which the participants viewed 12 weekly videos about different topics on healthy habits: (1) Motivation for change; (2) Nutrients, fiber and water; (3) Frequency of food; (4) Breakfast and between meals; (5) From the market to your mouth; (6) Circadian rhythm; (7) Exercise recommendations; (8) False eating and exercise myths; (9) Body composition reference values; (10) Chronic diseases; (11) Nutritional strategies; and (12) Exercise strategies.

Three weeks after starting this first intervention, the nutritional intervention was carried out, consisting of nine nutritional workshops of 90 min duration, face-to-face and weekly, with nutritionists. In these workshops, activities were carried out, such as planning the weekly menu, planning Mediterranean diet meals, organizing the macronutrient intake following the Mediterranean diet pattern, and reinforcing the nutritional information of the videos.

Once the nutrition intervention was completed and coinciding with the home lockdown ordered by the government of Spain to stop the expansion of the COVID-19 outbreak, the PA intervention was carried out. This intervention, telematically-supervised in real-time, lasted six weeks, with 18 sessions of 60 min each, with a frequency of three sessions per week, combining strength and resistance exercises in each session, following 2020 WHO recommendations [26].

After a 10-min warm-up of mobility exercises, training sessions consisted of a 40-min combination of strength training and moderate-intensity aerobic training. The aerobic training consisted of walking around the house, stationary bike, or jogging at home. The strength training consisted of a 2–3 sets circuit of 7–8 strength exercises for major muscle groups, performing 12–15 repetitions for each exercise, working mainly with self-loads due to the low material available to the participants at home, with a rest time of 30 s between exercises, and 1 min between sets. Both aerobic and strength training were performed at an intensity of 7–8 on the Borg Perceived Exertion Scale (RPE). Finally, a 10-min cool down was performed, consisting of flexibility exercises.

The nutrition intervention was directed and supervised in person by two nutritionists per participant, and the physical exercise intervention was telematically supervised in real-time by two trainers for each participant.

Once the physical exercise intervention ended, the IG received some PA and nutrition recommendations to maintain their new healthy habits for six months. The objective was to measure long-term adherence to the program (Figure 1).

Throughout this process, the CG continued with its ordinary activities without monitoring.

### 2.7. Place of the Intervention and Times of Assessments

The nutritional intervention was carried out at the facilities of the Universidad Europea de Madrid, during the employees’ workday. The physical exercise intervention was carried out using a telematics platform, as the subjects were at home to the COVID-19 lockdown. The initial assessments (T1) were performed before starting the interventions, and the final assessments (T2) were performed six months after the end of the interventions.

### 2.8. Statistical Analysis

All the results were analyzed by protocol and intention-to-treat analysis (ITT). The distribution and normality of the data were analyzed with the Shappiro–Wilk and Levene tests and with P-P and Q-Q plots. Data are expressed as mean ± SD. The independent T test and the Mann–Whitney U-test were used to compare the differences between both groups (CG and IG) before the intervention, with the aim of evaluating the homogeneity of the groups. A two-way analysis of variance (ANOVA) with repeated measures was conducted, to determine the effects of the intervention. To assess the effects of the intervention and minimize the risk of type I error, only interactions between time groups were considered. The level of statistical significance was set at *p* < 0.05. Eta partial squared (η^2^_p_) was used as a measure of effect size [61], considering 0.01 a low effect size, 0.06 a moderate effect size, and 0.14 a large effect size [62]. All statistical analysis was performed with SPSS 27.0 (IBM, Armonk, NY, USA).

## 3. Results

### 3.1. Recruitement

**Figure 2:**
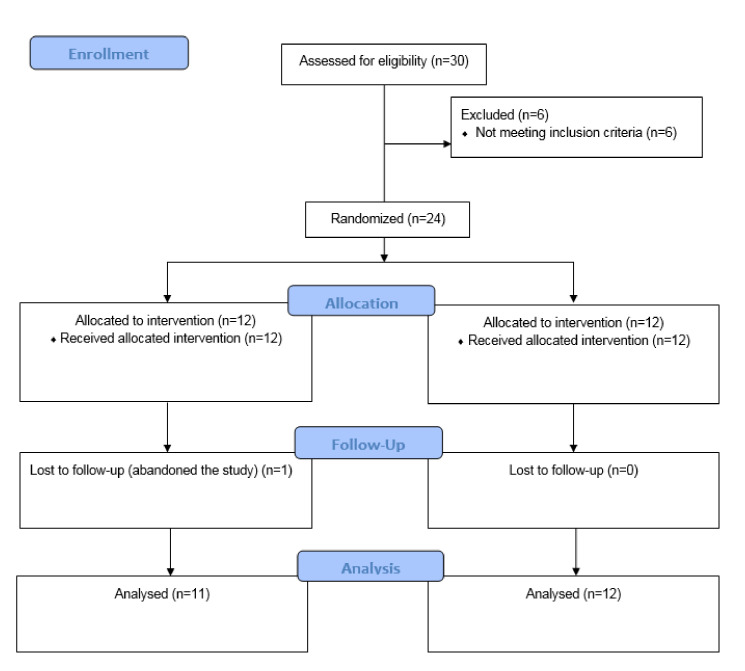
CONSORT Flow diagram of a lifestyle intervention during the COVID-19 pandemic.

Of 30 initially recruited adults, six (20%) did not meet the inclusion criteria. The remaining 24 subjects were randomly assigned into two groups, IG (*n* = 12) and CG (*n* = 12). There was one dropout in the IG so the final analysis was performed on 12 CG and 11 IG subjects, as is shown in the flow diagram (Figure 2). This study ended six months after the supervised exercise and nutrition intervention to assess long-term adherence to the intervention.

### 3.2. Description of the Sample

In the IG, 58% of the subjects were women and 42% were men, while in the CG, 83% were women and 17% were men. The mean age of the IG was 42.78 ± 6.88 years, the bodyweight was 74.98 ± 15.68 kg, the height was 169.23 ± 8.04 cm, and the BMI was 25.82 ± 3.70 kg/m^2^; while the mean age of the CG was 40.46 ± 7.77 years, the weight was 66.28 ± 12.82 kg, the height was 164.23 ± 8.04 cm, and the BMI was 24.53 ± 4.19 kg/m^2^. There were no significant differences in these variables between the two groups.

### 3.3. Lifestyle

As a result of adherence to the intervention, compared to the CG, the IG participants significantly improved their lifestyle in the time x group interaction, both in the total score of the HPLP II questionnaire (121.27 ± 12.54, vs. 141.73 ± 17.43 respectively; *p_txg_* = 0.03; *η^2^p* = 0.22), and in the categories of Health Responsibility (17.00 ± 2.61, vs. 21.36 ± 4.25; *p_txg_* = 0.02, *η^2^p* = 0.22), Physical Activity (14.00 ± 4.27, vs. 19.81 ± 4.31; *p_txg_* = 0.02, *η^2^p* = 0.22) and Nutrition (21.82 ± 2.68, vs. 22.25 ± 4.71; *p_txg_* = 0.02, *η^2^p* = 0.23), with a large effect size for these four variables. Regarding Adherence to the Mediterranean diet, both groups progressed from low adherence (MEDAS score ≤ 7) to medium adherence (MEDAS score 8–9), with no significant differences between groups (*p_txg_* = 0.16). Regarding PA levels, both groups showed an increase, progressing from low to moderate levels [26], without observing significant differences between them (*p_txg_* = 0.52). Finally, the number of hours sitting decreased 2.5 h in the IG, although without significance in the time x group interaction (*p_txg_* = 0.09) (Table 1).

### 3.4. Health-Related Quality of Life

The IG showed an improvement trend in all HrQoL, being clinically significant in the Physical Component Summary (>4 points) but not in the Mental Component Summary (<4 points) [60]. However, in the time x group interaction these improvements were not statistically significant in any of the eight categories of HrQoL: Physical Function (*p_txg_* = 0.60, *η^2^p* = 0.01), Role Physical (*p_txg_* = 0.13, *η^2^p* = 0.11), Bodily Pain (*p_txg_* = 0.21, *η^2^p* = 0.07), General Health (*p_txg_* = 0.10, *η^2^p* = 0.12), Vitality (*p_txg_* = 0.08, *η^2^p* = 0.14), Social Functioning (*p_txg_* = 0.45, *η^2^p* = 0.03), Mental Health (*p_txg_* = 0.73, *η^2^p* = 0.01), Role Emotional (*p_txg_* = 0.79, *η^2^p* < 0.01), Physical Component Summary (*p_txg_* = 0.12, *η^2^p* = 0.11), and Mental Component Summary (*p_txg_* = 0.75, *η^2^p* = 0.01). The effect size was large for Vitality, and Moderate for the Role Physical, General Health, and the Physical Component Summary (Table 1).

### 3.5. Anthropometric Variables

Bodyweight (*p_txg_* = 0.17) and BMI (*p_txg_* = 0.16) did not change after the intervention.

#### Compliance with the Exercise and Nutrition Intervention

Compliance with the intervention was high among the IG, with a mean attendance at the training sessions of the physical exercise intervention of 92% and a mean attendance at the workshops of the nutritional intervention of 84%. There were no adverse effects caused by the intervention.

## 4. Discussion

Six months after this multi-component intervention based on nutritional workshops and telematically-supervised aerobic and strength exercise, the IG improved adherence to a healthier lifestyle, as indicated by the total score of the HPLP II questionnaire, in which they progressed from a medium score to a high score. Likewise, the IG showed significant improvements in the time x group interaction, with a large effect size in the sub-categories of Health Responsibility, Physical Activity, and Nutrition. On the other hand, when we analyzed adherence to the Mediterranean diet six months after the intervention, no significant differences were observed between the groups, as both groups reached scores of moderate adherence (MEDAS score of 8–9). Furthermore, the number of hours sitting per day and the PA levels did not show a significant variation between groups. However, the IG group showed a reduction in the daily sitting time but not the CG. Possibly, the exceptional situation of the COVID-19 lockdown, the mobility restrictions, and the novel context of teleworking influenced these results. In addition, the fact that both groups analyzed were in a state of predisposition to change, according to the study’s inclusion criteria, could have caused the CG to make changes in their lifestyle, not finding significant differences regarding HrQoL.

According to a recent systematic review, many studies report an increase in the PA levels after a telematic intervention, but very few assess the adherence to these habits six months later, without achieving beneficial results [46]. Regarding sedentary behavior, a recent meta-analysis reported that telematic interventions achieve a reduction of one hour of sitting time per day, although six months later, the effects are practically nil, not achieving adherence to healthy habits [47]. Regarding adherence to the Mediterranean diet, many studies assess the effectiveness of Mediterranean diet interventions on cardiovascular health, but very few studies perform a long-term post-intervention follow-up of this variable. Only one randomized clinical trial carried out in type 2 diabetics has been reported, in which, after achieving a post-intervention increase in the adherence to this type of diet, they managed to maintain these improvements after nine months [63]. Finally, the only study using the HPLP II questionnaire that performed a follow-up after a lifestyle intervention reported significant improvements in the total score, but only three months had elapsed past the intervention, which is a short-term follow-up [64].

To date, there are no lifestyle interventions during the COVID-19 pandemic in the workplace described in the literature, as there is only one observational study [6]. We provide a randomized clinical trial with a physical exercise and nutrition intervention and a long-term assessment of adherence to healthy habits.

The participants of this study were middle-aged university employees with adequate body weight and without diagnosed associated diseases. They had an improvable health-promoting lifestyle score [56], low PA levels, less than 350 METS-min per week [26], low adherence to the Mediterranean diet, high values of sedentary behavior, 8 h a day sitting [15,17,26,51,65], and a HrQoL below average compared to the reference values for their age range [66].

The IG achieved a significant improvement regarding diet, analyzed with the HPLP II questionnaire, with a large effect size. It implied the acquisition of healthy habits such as “following a diet low in saturated fat”, “limiting the consumption of sugars and sweets”, “eating 2–4 servings of fruit and 3–5 servings of vegetables a day”, or “limiting the salt intake”. However, when analyzing adherence to the Mediterranean diet using the MEDAS questionnaire, both groups increased their adherence to this diet, as did the general population in many countries during the COVID-19 pandemic [7,8], progressing from a low to a medium adherence. However, the IG achieved a clinically significant improvement [57] on the MEDAS score of 40%, compared to 26% for the CG, with a moderate effect size, reaching a score close to 10, which corresponds to the high adherence category [52]. This improvement was not statistically significant in the time x group interaction, possibly due to the sample size and the state of predisposition to change in which all the subjects were at the beginning of the study. It should be noted that very few studies of high methodological quality are reported with Mediterranean diet interventions carried out in the workplace. Only two randomized clinical trials [40,44] consisting of Mediterranean diet interventions lasting for 18–24 months, managed to increase the consumption of monounsaturated fatty acids and polyunsaturated fatty acids, and to reduce the consumption of total cholesterol and saturated fats. Nevertheless, they did not use the MEDAS questionnaire, and they did not perform a long-term post-intervention follow-up to assess adherence to the intervention [52].

The IG achieved a significant improvement in their lifestyle in terms of PA, measured with the HPLP II questionnaire, with a large effect size in the time x group interaction. This improvement involved the acquisition of habits such as “follow an exercise program”, “perform vigorous PA for at least 20 min 3 times a week”, or “perform light to moderate-intensity PA for at least 30–45 min 5 times a week”. However, when performing a quantitative analysis of the PA levels expressed in weekly METS-min through the GPAQ questionnaire [58], both groups increased this variable, the increase being 305% for the IG, and 221% for the CG, progressing from low to medium PA levels [26], without finding significant differences in the time x group interaction, and with a low effect size. The progressive elimination of mobility restrictions and lockdown, in subjects who were in a phase of predisposition to change, possibly caused everyone to increase their PA levels. However, very few randomized controlled trials performed in the workplace achieve a significant quantitative increase in the PA levels [42,48,67,68,69], and only one study maintains these results six months post-intervention [48]. The difference concerning our study is the unique context of a pandemic in which our participants have found themselves.

In previous studies carried out during the COVID-19 pandemic, there have been reported increases of 23.8–28.6% in the number of hours sitting per day [23,45]. Yet, in our study, maintenance of this variable was observed in the CG. The IG participants reduced their daily sitting time by 33%, achieving a reduction of 2.5 h per day, which is considered clinically significant, with a moderate effect size (*η^2^p* = 0.13). These results were not statistically significant, possibly due to the size of the sample. Other studies performed in the workplace have reported significant reductions post-intervention regarding sedentary behavior, but without assessing long-term adherence [43]. Bodyweight and BMI did not change, which may be because the physical exercise intervention had a large component of strength exercise and possibly increased muscle mass, as occurred with the interventions described in the studies by der Schoenfeld et al [70].

Regarding HrQoL, the IG showed an improvement trend in all the variables, being clinically relevant in the Physical Component Summary (>4 points), with a moderate effect size, but not in the Mental Component Summary [60]. This could be due to the difficult circumstances that we have faced during the COVID-19 pandemic, which has negatively affected our mental health [71]. The highest improvements were found in the domains of Role Physical, General Health, and Vitality, with a moderate effect size. However, there were no statistically significant differences between groups, so these improvements cannot be attributed to the intervention.

According to the Transtheoretical Model of Behavior change (TMBC) [72], the optimal stage for performing a lifestyle intervention is the contemplation stage when the subjects are ready to face lifestyle changes [73]. The TMBC has turned out to be an effective strategy for promoting lifestyle habit changes [74]. The fact that all the subjects of our study were in the contemplation stage possibly led the CG to make their own lifestyle changes, and for this reason, we did not find statistically significant differences in some variables in the interaction time x group. Our results cannot be extrapolated to all populations that are not in the contemplation stage.

However, adherence to the lifestyle changes achieved in this study is an effective primary prevention strategy for preventing NCDs [24]. One of the strengths of this program is that the IG showed high compliance with the intervention.

We believe that lifestyle programs or interventions should be promoted in the workplace, especially during the COVID-19 pandemic, as the general population has reduced its PA levels. Perhaps, for greater benefits, exercise programs should be of a longer duration [36,37,38,39].

Regarding the limitations of the study, the researcher who analyzed the statistical data was not blinded, and we did not control the caloric intake of the subjects.

## 5. Conclusions

**Figure 3:**
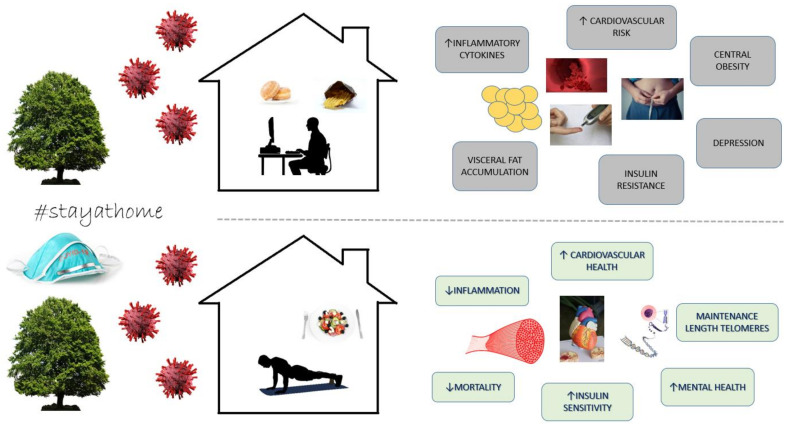
Lifestyle during the COVID-19 pandemic and its repercussions on health [9,10,11,12,13,22,23,24,25,28,29,30].

Despite the lockdown and the mobility restrictions caused by the COVID-19 pandemic, this supervised physical exercise and nutrition intervention performed on university employees achieved adherence to a healthier lifestyle (Figure 3). The participants increased their PA levels, reduced their daily sitting time by 2.5 h, and improved their HrQoL in the Physical Component Summary by more than 4 points, which is of great clinical relevance (Figure 3).

## Figures and Tables

**Table 1:** Comparison of lifestyle variables and health-related quality of life in the two assessment moments.

Variables	Group	T1	T2	*p*-Value Time	*p*-Value Group	*p*-Value txg	η^2^p txg
HPLP II							
Health-Promoting Lifestyle (total score)	IG CG	121.27 ± 12.54 123.67 ± 15.33	141.73 ± 17.43 131.58 ± 13.83	<0.001	0.50	* 0.03	0.22
Health Responsibility	IG CG	17.00 ± 2.61 18.08 ± 4.52	21.36 ± 4.25 19.67 ± 4.16	<0.001	0.85	* 0.02	0.22
Physical Activity	IG CG	14.00 ± 4.27 13.33 ± 3.60	19.81 ± 4.31 14.83 ± 4.45	<0.001	0.07	* 0.02	0.22
Nutrition	IG CG	21.82 ± 2.68 22.25 ± 4.71	26.64 ± 2.84 24.08 ± 4.70	<0.001	0.49	* 0.02	0.23
Spiritual growth	IG CG	24.64 ± 2.80 26.50 ± 3.75	27.27 ± 2.90 28.33 ± 4.10	0.001	0.28	0.52	0.02
Interpersonal Relations	IG CG	27.18 ± 3.79 27.33 ± 3.85	27.67 ± 3.91 27.42 ± 3.87	0.69	0.98	0.78	0.004
Stress management	IG CG	16.64 ± 2.77 16.17 ± 2.41	19.00 ± 3.46 17.25 ± 2.80	0.01	0.30	0.28	0.06
MEDAS							
Adherence to the Mediterranean diet	IG CG	7.00 ± 1.41 7.08 ± 1.08	9.82 ± 1.60 8.92 ± 2.02	<0.001	0.47	0.16	0.09
GPAQ							
Physical activity levels (METS-min per week)	IG CG	327.27 ± 258.96 316.67 ± 367.48	1327.27 ± 1046.15 1016.67 ± 1039.26	0.001	0.49	0.52	0.02
Daily sitting time (min)	IG CG	463.64 ± 180.18 540.00 ± 209.41	312.73 ± 150.80 559.17 ± 259.32	0.18	0.03	0.09	0.13
SF-36							
Physical Component Summary	IG CG	49.06 ± 5.04 51.78 ± 7.20	54.51 ± 4.02 50.25 ± 8.52	0.37	0.65	0.12	0.11
Mental Component Summary	IG CG	51.43 ± 8.24 40.60 ± 12.76	53.07 ± 5.99 43.70 ± 11.73	0.32	0.01	0.75	0.01

T1, initial assessment; T2, final assessment; IG, intervention group; CG, control group; η^2^p txg: effect size time x group; * (bold), *p* < 0.05; Differences between group, time and group x time interaction were evaluated using two-way repeated measure ANOVA. Significance was set at < 0.05.

## Data Availability

Data available upon request due to ethical and privacy restrictions.
